# Mechanism of homocysteine-mediated endothelial injury and its consequences for atherosclerosis

**DOI:** 10.3389/fcvm.2022.1109445

**Published:** 2023-01-16

**Authors:** Deqiang Yuan, Jiapeng Chu, Hao Lin, Guoqi Zhu, Jun Qian, Yunan Yu, Tongqing Yao, Fan Ping, Fei Chen, Xuebo Liu

**Affiliations:** Department of Cardiology, Tongji Hospital, School of Medicine, Tongji University, Shanghai, China

**Keywords:** homocysteine, endothelial cells, hyperhomocysteinemia, atherosclerosis, inflammation

## Abstract

Homocysteine (Hcy) is an intermediate amino acid formed during the conversion from methionine to cysteine. When the fasting plasma Hcy level is higher than 15 μmol/L, it is considered as hyperhomocysteinemia (HHcy). The vascular endothelium is an important barrier to vascular homeostasis, and its impairment is the initiation of atherosclerosis (AS). HHcy is an important risk factor for AS, which can promote the development of AS and the occurrence of cardiovascular events, and Hcy damage to the endothelium is considered to play a very important role. However, the mechanism by which Hcy damages the endothelium is still not fully understood. This review summarizes the mechanism of Hcy-induced endothelial injury and the treatment methods to alleviate the Hcy induced endothelial dysfunction, in order to provide new thoughts for the diagnosis and treatment of Hcy-induced endothelial injury and subsequent AS-related diseases.

## 1. Introduction

Hyperhomocysteinemia (HHcy) is an important risk factor for atherosclerosis (AS) and is defined as fasting plasma homocysteine (Hcy) higher than 15 μmol/L (1). Hcy is formed during the conversion of an essential amino acid methionine to cysteine, the factors that affect the level of Hcy in plasma include genetics, nutrition, age, sex, drugs, disease state. Hcy as an intermediate amino acid affects many cellular biological processes, such as cellular methylation status, cell metabolism, and cell injury.

Endothelial cells (ECs) are located in the innermost layer of blood vessels and form the vascular intima, which is important for maintaining vascular homeostasis and normal blood circulation. Pro-atherosclerotic risk factors in the blood can damage the endothelium, which acts as the initiation of AS and causes its progression. In recent years, numerous studies have shown that Hcy can damage the endothelium through various mechanisms, which may be a key way for it to promote AS-related diseases (2). Although the specific mechanism of Hcy injury to the endothelium is still not fully clear, it may be related to the induction of inflammation and cell death, interference with nitric oxide (NO) production, reactive oxygen species (ROS) accumulation and oxidative stress, cellular hypomethylation, protein homocysteinylation, and abnormal lipid metabolism (2). Hcy can cause intimal damage through these mechanisms and aggravate the progression of AS, and more in-depth mechanisms need to be studied. In terms of treatment, besides dietary therapy and supplementation of vitamin B12/B6 and folate to reduce plasma Hcy level, many drugs that can alleviate Hcy-induced endothelial damage have been studied, such as melatonin (3), estrogen (4), some lipid-lowering (5), and hypoglycemic (6) drugs. It is of great scientific and clinical significance for the prevention and treatment of endothelial injury and subsequent AS caused by HHcy to further explore the mechanism and mitigation methods of Hcy damage to the endothelium. This review mainly summarizes the mechanism of Hcy damage to ECs and related treatment, aiming to provide some new perspectives for research and clinical practice in this direction.

## 2. Homocysteine metabolism

**FIGURE 1:**
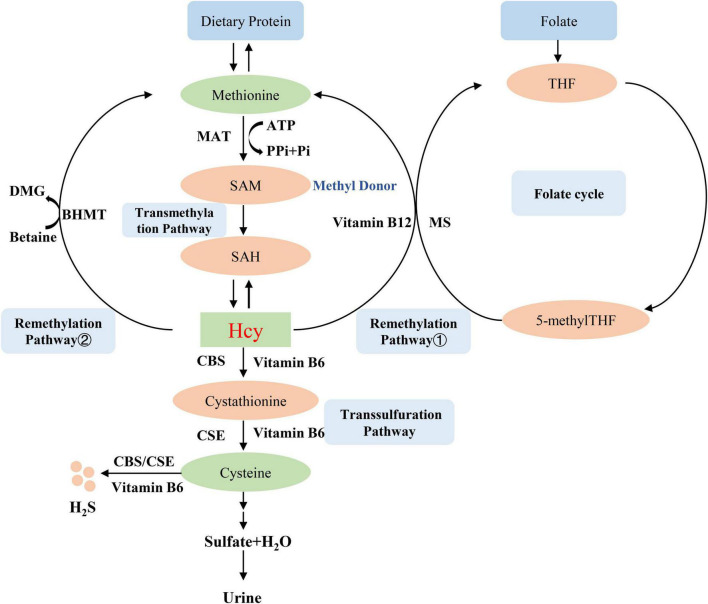
Homocysteine metabolism. MAT, methionine adenosyltransferase; SAM, S-adenosylmethionine, AdoMet; SAH, S-adenosylhomocysteine, AdoHcy; CBS, cystathionine β-synthase; BHMT, betaine-homocysteine methyltransferase; MS, methionine synthase; THF, tetrahydrofolate; 5-methylTHF, 5-methyltetrahydrofolic acid; CSE, cystathionine-gamma-lyase.

Homocysteine is an intermediate amino acid formed during the conversion from an essential sulfur-containing amino acid methionine to cysteine (7). The source of methionine is dietary protein, and the liver is considered to play an important role in the methionine and Hcy metabolism because of its full complement of related enzymes to regulate plasma Hcy level (8). Hcy is produced *via* transmethylation and removed through either the remethylation or the trans-sulfuration pathways (9). First, methionine is converted into the high-energy sulfonium compound S-adenosylmethionine (SAM, AdoMet), which is an important methyl donor for nearly all cell transmethylation process (10), the reaction is catalyzed by methionine adenosyltransferase (MAT) and provided the adenosyl moiety by adenosine triphosphate (ATP) (11). Next, SAM is demethylated to form S-adenosylhomocysteine (SAH, AdoHcy), which is then hydrolyzed to adenosine and Hcy (9). Hcy then experiences the transsulfuration pathway to form cysteine and glutathione or the remethylation cycle to back to methionine (12). The transsulfuration pathway tends to occur through upregulating the vitamin B6-dependent enzyme, cystathionine β-synthase (CBS) and cystathionine-gamma-lyase (CSE), and downregulating the remethylation pathway in the situations of excess methionine (13). Therefore, the lack of CBS will lead to the abnormal accumulation of Hcy and the occurrence of related diseases such as homocystinuria, homocysteinemia, and hypermethioninemia (14). The cysteine that is formed from Hcy by transsulfuration is finally oxidized to the sulfate and is excreted in the urine (15). On the other hand, Hcy is remethylated to methionine by using 5-methyltetrahydrofolic acid (5-methylTHF) or betaine as a methyl donor (16). These two different reactions are more likely to occur in methionine deficiency. In the first reaction, Hcy is catalyzed by methylcobalamin-containing methionine synthase (MS) to form methionine and tetrahydrofolate (THF) *via* receiving methyl from 5-methylTHF (16). Thus, Hcy metabolism generates cross-link with intracellular folate metabolism (17), which is also an important reason for folate level affect Hcy metabolism. In the second reaction, betaine-dependent remethylation backs Hcy to methionine by betaine-homocysteine methyltransferase (BHMT) (18). Several B vitamins exert important role in Hcy metabolism, vitamin B12 is the cofactor for MS (19), vitamin B6 is the coenzyme for CBS (20), so these B vitamins deficiencies can lead to Hcy accumulation. The mechanism of Hcy metabolism described above is shown in Figure 1.

Intracellular concentration of Hcy is often under tight control by the above-mentioned reactions and by controlling its export from the cell (2). Plasma Hcy can easily act on ECs located in the innermost layer of blood vessels, and has significant effects on ECs function (21). There are three different forms of plasma Hcy, which including free Hcy, protein-bound Hcy and oxidized forms of Hcy (22). The Hcy concentration currently measured in the clinic is representative of the total plasma Hcy concentration. The normal range for plasma Hcy is 5–15 μmol/L measured in the fasting state, so HHcy is defined as plasma Hcy higher than 15 μmol/L (23). And HHcy is categorized into three classes as mild, moderate and severe HHcy with plasma HCy levels ranging from 15 to 30, 31 to 100, and >100 μmol/L, respectively.

## 3. Causes of hyperhomocysteinemia

**FIGURE 2:**
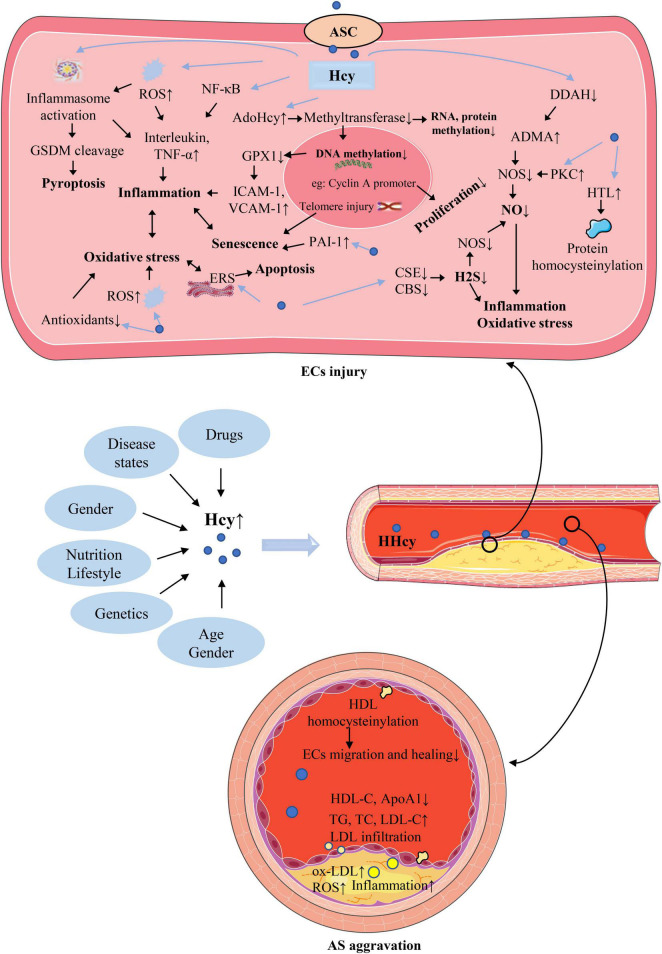
The causes of hyperhomocysteinemia (HHcy) and the mechanism of homocysteine (Hcy)–mediated endothelial cells (ECs) injury and its consequences for atherosclerosis (AS). The abbreviations in this figure are shown in the “Glossary” section.

Plasma Hcy level is affected by a variety of factors, as shown in Figure 2. Such as genetics, nutrition, age, sex, drugs, disease states, which are described in detail below. The exploration of the causes of elevated plasma Hcy level is of great significance for the treatment of HHcy and various injuries caused by it.

### 3.1. Genetics

Severe elevations of Hcy concentration in plasma is rare and often caused by homozygous mutations in enzymes involved in its metabolism, such as MS, methylene tetrahydrofolate reductase, and CBS. Numerous studies have investigated the changes in plasma concentration of Hcy and related disease progression in experimental animals after CBS knockout. The researchers used CRISPR/Cas9 to knock out the CBS gene in rabbits and found that the plasma Hcy level in knockout rabbits (50.73 μmol/L) was almost twice as high as that in controls (27.93 μmol/L) (24). And severe HHcy was observed in CBS^–/–^ (289 ± 58 μM) but not in CBS^±^ or control mice (<10 μM) after knocking out CBS in mice (25). The clinical manifestations of homozygous HHcy often include psychiatric symptoms and abnormalities of the hair, skin, joints, bones, and cardiovascular system. However, HHcy caused by heterozygous mutations in related enzymes is often asymptomatic, with only moderately elevated or normal plasma Hcy (26).

### 3.2. Nutrition and lifestyle

As mentioned above, B vitamins and folate exert important role in the metabolism of Hcy. Therefore, plasma Hcy concentration can be significantly increased in the deficiency of essential cofactor Vitamin B12/B6 or cosubstrate folate (27). Asian populations are prone to insufficient folate intake due to dietary and cooking habits, which may partly account for incidence of folate deficiency and HHcy in Asian populations is much higher than that in Western populations (28, 29). The incidence of HHcy in American society is just 5–7%, in Chinese 27.5% and in Indians 52–84% (30). Inappropriate lifestyle, such as high intake of coffee (31), alcohol, obesity and smoking can also lead to HHcy (32), which may be related to vitamin malabsorption.

### 3.3. Age

Plasma Hcy is generally believed to increase with age (33). The specific mechanism of this phenomenon is still unclear. The possible reasons include the reduction of Hcy metabolizing enzyme activity, the impairment of renal function, hormonal changes, and the reduction of vitamin B12/B6 level as cofactor (34).

### 3.4. Gender

Plasma Hcy concentration in premenopausal women is typically 20% lower than in men of similar age. This may be related to the higher creatinine concentration and more muscle mass in men (34). However, female menopause is comparable to male plasma Hcy level. This suggests that Hcy is related to the change of estrogen level, and the decrease of Hcy level by estrogen may be related to the increase of CBS activity (35).

### 3.5. Drugs

Some lipid-lowering drugs such as fibrates (36) and niacin can increase plasma Hcy. Mechanistically, fenofibrate may significantly increase plasma Hcy levels by reducing renal function (37). Therefore, the benefits of treating elevated cholesterol concentrations with these drugs should be weighed against the possible long-term risks of elevated Hcy. Methotrexate inhibits the conversion of dihydrofolate to tetrahydrofolate with physiological activity by inhibiting dihydrofolate reductase (DHFR), thereby increasing plasma Hcy (38, 39).

### 3.6. Disease states

Studies have shown that plasma Hcy level is elevated in some disease states, such as various cancers (40), psoriasis (41), hypothyroidism (42), diabetes (43), and renal dysfunction (16). Among them, the disease state most closely related to HHcy is chronic renal failure. Plasma Hcy level is positively correlated with creatinine level (44), and the specific mechanism remains to be further studied. The possible mechanism is that chronic renal failure inhibits the activity or inactivates the key enzymes of Hcy metabolism, such as CBS (45) and BHMT (46), resulting in the inhibition of Hcy metabolism and accumulation of Hcy. HHcy occurs in the early stage of chronic renal failure and further increases with the progression of renal failure. Elevated Hcy can further aggravate renal injury by inducing oxidative stress (47), insufficient autophagy and renal aging (48), forming a negative feedback loop. Elevated plasma Hcy level in patients with malignant tumors may be related to changes in methionine metabolism in tumor cells (49). The relationship between diabetes and HHcy is relatively complex. It is generally believed that secondary pathological changes caused by diabetes, such as renal dysfunction, often lead to elevated plasma Hcy level, and the impact of diabetes itself on Hcy needs further study (50). The possible mechanism is that glucose metabolism disorder and Hcy metabolism worsen each other, resulting in the increase of Hcy level (32, 51).

## 4. Endothelial dysfunction and atherosclerosis caused by hyperhomocysteinemia

Vascular endothelium is a homeostatic barrier to circulating blood and vessel walls like a gatekeeper of cardiovascular health (52). The vascular intima plays an important role in the homeostasis regulation of the vascular wall, including vascular tone, coagulation, inflammation, and permeability (53). As the innermost layer of the blood vessel wall, ECs are directly exposed to circulating blood, so they are easily injured by various risk factors and lead to their dysfunction and endothelial barrier damage (54). Impaired endothelial function and barrier can lead to a series of cascade reactions, such as inflammatory response (55), monocyte recruitment (56), plaque formation (57), structural remodeling (58), thrombosis (59). The results of cultured ECs and animal models of HHcy indicate that Hcy can injury ECs and cause endothelial dysfunction (60, 61). Several studies have confirmed that plasma Hcy concentration is positively correlated with AS. Clinical studies have shown that plasma Hcy level in patients with coronary artery disease is significantly higher than that in angiographic normal controls (62), and high plasma Hcy level with only 12% of the upper limit of normal are associated with a 3.4-fold increased risk of myocardial infarction (63). The above description is the newly proposed homocysteine theory of AS. H-type hypertension is a special type of hypertension combined with elevated plasma Hcy on the basis of hypertension. The risk of stroke caused by this kind of hypertension is obviously higher than that of simple hypertension (64), which may be related to Hcy damage to the vascular endothelium. This suggests that Hcy-damaged endothelium plays a central role in its promotion of vascular diseases. Therefore, exploring the mechanism of Hcy injury to vascular endothelium is of great significance for the diagnosis and treatment of AS-related diseases.

## 5. Mechanism of endothelial injury by homocysteine

Homocysteine can cause ECs damage through various intracellular mechanisms. Such as induction of inflammation and cell death, interference with NO production, ROS accumulation and oxidative stress, cellular hypomethylation. There is a complex interaction between these mechanisms, which leads to a series of reactions in the local and circulation of AS lesions. In addition, abnormal lipoprotein metabolism major as an extracellular mechanism also causes ECs damage and promotes AS progression. Protein homocysteinylation can cause endothelial damage through both intracellular and extracellular mechanisms. A brief illustration of these mechanisms is shown in Figure 2 and described in detail below.

### 5.1. Induce inflammation and cell death

Endothelial inflammation is a crucial driver of AS. When atherosclerotic risk factors act on ECs, damaged ECs secrete a large number of cytokines, adhesion molecules, and chemokines (65). These secreted factors recruit circulating monocytes to the site of endothelial injury and induce the transformation of monocytes into pro-inflammatory macrophages, which in turn develop into foam cells, promoting the formation of atherosclerotic plaques at the site of injury (66). Experimental studies on cultured ECs have shown that Hcy can induce inflammation by inducing a variety of inflammatory cytokines, such as interleukin (IL)-1β (67), IL-6 (68), IL-8 (69, 70), IL-18 (71), and tumor necrosis factor (TNF)-α (72), which may be due to the ROS accumulation, inflammasome activation, nuclear factor kappa-B (NF-κB) activation. Hcy can promote ECs senescence by upregulating plasminogen activator inhibitor-1 (PAI-1) (73), and telomere shortening and dysfunction may also be the cause of HCy-induced ECs senescence (74). ECs senescence further accelerates inflammation and endothelial injury by senescence-associated secretory phenotype (SASP). When the damage of Hcy to ECs is further aggravated, the cells may go toward death, resulting in severe endometrial damage. Numerous studies have shown that Hcy can cause various forms of ECs death, such as apoptosis (75), pyroptosis (76), and ferroptosis (77). Pyroptosis is a newly discovered form of programed cell death, which depends on gasdermin family proteins such as gasdermin D (GSDMD), GSDMB, GSDME. Both *in vivo* and *in vitro* studies have shown that Hcy can induce ECs pyroptosis and release of inflammatory factors such as IL-1β and IL-18 *via* caspase-1-dependent inflammasome activation through the accumulation of intracellular ROS (76). ECs death causes cell death on the one hand, and some inflammatory cell death forms, such as pyroptosis, can cause strong local and circulatory inflammatory responses and accelerate the progression of AS.

### 5.2. Interfere with NO production

Nitric oxide is a key signaling molecule in endothelium to act as vasodilator factor, generated by nitric oxide synthase (NOS) (78). Endothelial production of NO can inhibit multiple events during AS, such as inhibiting ECs activation, macrophage infiltration (79), foam cell formation/migration (80), platelet aggregation (81), inflammation (82), thrombosis (83), vascular wall remodeling (84), and mediating vasodilation (85). The mechanism of Hcy disturbance of NO synthesis is relatively complex, asymmetric dimethylarginine (ADMA) plays an important role in it, which is an endogenous inhibitor of NOS. Specifically, Hcy post-translationally inhibits dimethylarginine dimethylaminohydrolase (DDAH) activity, the enzyme that degrades ADMA (86). Therefore, Hcy can cause ADMA to accumulate and inhibit NO synthesis. Hcy can also inhibit NOS and reduce NO synthesis in ECs by activating protein kinase C (PKC). Reduced NO synthesis causes endothelial injury by aggravating oxidative stress and inflammation (87).

### 5.3. ROS accumulation and oxidative stress

Major producing systems of ROS in ECs include reduced form of nicotinamide adenine dinucleotide phosphate (NADPH) oxidase, xanthine oxidase, the mitochondrial electron transport chain, and uncoupled endothelial NOS (79). Moderate concentrations of ROS have important signaling functions, while excessive accumulation of ROS can defeat the antioxidant system, leading to oxidative stress. A large number of studies illustrate that oxidative stress plays an important role in Hcy-induced ECs dysfunction and AS. Specifically, Hcy can induce oxidative stress in ECs by mediating ROS production or impairing the antioxidant system (2). Hcy also can induce ECs NADPH oxidase upregulation to accumulate ROS (2). An important mechanism of Hcy-induced ECs injury is endoplasmic reticulum stress (ERS), which is a response to dangerous stimulation that leads to the accumulation of unfolded or abnormally folded proteins in the endoplasmic reticulum (ER) (88). Cell and animal model results found that Hcy upregulates ER oxidoreductin-1α (Ero-1α) expression by promoting binding of hypoxia-inducible factor-1α (HIF-1α) to the Ero-1α promoter and downregulates the antioxidant pathway mediated by ER glutathione peroxidase (GPX) 7 to induce ERS and disrupt ER homeostasis (89). Oxidative stress and ERS aggravate each other. When ECs suffer from oxidative stress, the redox equilibrium of ER is broken, thereby disrupting ER function and triggering ERS. Similarly, when ERS occurs, a large amount of ROS will be produced to aggravate oxidative stress. Moderate ERS is a protective response, but excessive ERS can lead to cellular dysfunction, inflammatory responses and apoptosis (90). Therefore, oxidative stress caused by Hcy is closely related to ECs inflammation and death. On the other hand, Hcy also can cause oxidative stress by inhibiting ECs non-enzymatic antioxidants and damages enzymatic antioxidants (2).

### 5.4. Hydrogen sulfide signaling pathway dysregulation

We already know that Hcy is metabolized to cysteine through the transsulfurization pathway, which is then decomposed into sulfate and excreted in urine. This pathway is regulated by two key enzymes, CBS and CSE, furthermore, these two enzymes can also use cysteine as a substrate and are dependent on pyridoxal-5’-phosphate to produce dihydrogen sulfide as shown in the Figure 1. Dihydrogen sulfide is also known as hydrogen sulfide (H_2_S) or sulfane (91, 92). H_2_S is considered to be an endogenously produced gasotransmitter that acts on various targeted signaling pathways to play an important role in vascular homeostasis (93, 94). Downregulation of the CSE/H_2_S pathway has an important pathological role in the development of AS (95). H_2_S is reported to regulate multiple endothelial functions such as angiogenesis, proliferation and migration (96). The study found that H_2_S pretreatment of ECs can improve mitochondrial function and cell viability after hypoxia treatment by activating extracellular regulated protein kinases (ERK) 1/2, as well as enhance ECs migration and angiogenesis, thereby protecting ECs from ischemia/reperfusion injury and gradually reducing cardiac damage (97). It has also been found that exogenous H_2_S treatment or CSE overexpression can rescue high glucose-induced ECs migration impairment by upregulating microRNA (miR)-126-3p (98). Although the role of H_2_S on inflammation is complex, it is very noteworthy that recent studies have suggested that H_2_S can inhibit ECs inflammation (99), such as inhibiting NF-κB pathway and scavenging ROS (2). Interestingly, H_2_S also has a heterotypic interaction with another important signaling molecule, NO, for example, H_2_S can promote endothelial NO production *via* activating NOS phosphorylation (100). The complex interaction between H_2_S and NO might serve as an important regulator for endothelial homeostasis.

Hyperhomocysteinemia can cause downregulation of CSE and CBS, thereby causing production of H_2_S is injury (2). Lack of H_2_S protection in ECs can cause endothelial dysfunction and damage, and gradually lead to HHcy-related vascular disease (96). The study has shown that exogenous H_2_S treatment can alleviate Hcy-induced endothelial dysfunction by inhibiting mitochondrial toxicity (101). Therefore, Hcy can impair endothelial homeostasis by impairing the H_2_S pathway, and maintaining the normal function of this pathway is important for Hcy-induced endothelial dysfunction.

### 5.5. Cellular hypomethylation state

S-adenosylmethionine, formed when methionine is converted into Hcy, is the methyl donor for almost all transmethylation reactions of DNA, RNA, protein and other components in cells (102, 103). Therefore, the methylation reactions of various cellular components can be significantly affected by Hcy metabolism. It is worth mentioning that, hydrolysis of S-AdoHcy to Hcy is invertible, and the synthesis of S-AdoHcy from SAM/AdoMet is thermodynamically more favored (104). The reaction toward hydrolysis direction because of rapid clearance of Hcy by cellular export and metabolic conversion under normal conditions. However, When Hcy levels are abnormally elevated, which can cause elevated AdoHcy. Importantly, AdoHcy is a critically endogenous inhibitor of cellular methyltransferases, HHcy is easy to form hypomethylating environment, which further impairs methylation reactions related to intimal homeostasis (105). ECs hypomethylation can reduce aquaporin-1 levels, leading to impaired water permeability and endothelial dysfunction (106). ECs hypomethylation can also upregulate the expression of the adhesion molecules intercellular adhesion molecule-1 (ICAM-1) and vascular adhesion molecule-1(VCAM-1) by suppressing GPX1 protein expression, causing ECs inflammation and injury (107). The epigenetic study has found that Hcy induces DNA hypomethylation of the cyclin A promoter by downregulating the expression of DNA methyltransferase 1 (DNMT1) to inhibit endothelial progenitor cells (EPCs) proliferation (108). These results suggest that Hcy-induced hypomethylation of DNA, RNA, protein in ECs leads to endothelial injury by impairing water permeability, inducing inflammation, and inhibiting proliferation.

### 5.6. Protein homocysteinylation

Homocysteinylation mainly targets proteins and is classified as S-homocysteinylation and N-homocysteinylation, N-homocysteinylation is mainly discussed here. When plasma Hcy level is elevated, N-homocysteinylation of numerous proteins in cells occurs due to the interaction between the highly reactive homocysteine thiolactone (HTL) and lysine residues of a target protein. Also, when HHcy is present in atherosclerotic patients, protein homocysteinylation is enhanced (22). HTL is a cyclic thioester generated during protein biosynthesis because of error-editing reaction of Hcy with methionyl-tRNA synthetase (MetRS) (7). The N-homocysteinylation of proteins can lead to abnormal protein structure and biochemical function, which is closely involved in the injury of vascular intima caused by HHcy (109). Cellular protein quality control (PQC) is an important event in maintaining cellular homeostasis to maintain proteome integrity and cell viability, which include unfolded protein response (UPR), autophagy, ubiquitin-proteasome system (UPS), and chaperones (110–113). Numerous studies suggest that HHcy can damage PQC by activating UPR, impairing autophagy, and reducing chaperone level (114).

High-density lipoproteins is considered to have endothelial repair function, and then it was found that when HDL is homocysteinylated by N-homocysteine, it reduces ECs migration and attenuates HDL-mediated endothelial healing compared with control (109). One study found that angiotensin-converting enzyme (ACE) could be directly homocysteinylated by Hcy, which enhances ACE reactivity toward angiotensin II (ANG II)-NADPH oxidase-superoxide-dependent endothelial injury (115). Therefore, finding more proteins with specific and sensitive homocysteine changes in endothelial injury is of great value for the diagnosis and treatment of AS.

### 5.7. Abnormal lipoprotein metabolism

Some metabolic changes outside ECs caused by HHcy can also damage the endothelium and promote AS. Abnormal lipid and lipoprotein metabolism is an important risk factor for AS (116). Lipoproteins such as Low-density lipoproteins (LDL) and high-density lipoproteins (HDL) are mainly responsible for the transport of lipids such as cholesterol and triglycerides (117). Several clinical and experimental studies have shown that HHcy affects lipid metabolism. A clinical study involving 7,898 subjects showed that plasma Hcy was negatively associated with high-density lipoprotein cholesterol (HDL-C) and apolipoprotein A1 (ApoA1) and positively associated with triglyceride (TG) (118). Moreover, a study (24) has found that the blood lipid levels of HHcy rabbits are significantly higher than that of the controls, the TG level showed almost 52-fold increase (3746.7/71.31 mg/dL). The total cholesterol (TC) (540.8 mg/dL) and low-density lipoprotein cholesterol (LDL-C) (72.02 mg/dL) in HHcy rabbits were almost 4.4-and 2.5-fold higher that in WT controls, respectively. These provides some evidences for HHcy disordered lipid metabolism. HDL mainly transports cholesterol from peripheral tissues such as blood vessels to the liver for metabolic clearance, so plasma HDL has an anti-atherosclerotic effect. However, the reduction of HDL in HHcy patients indicates that the reverse transport of cholesterol is impaired, thereby disturbing lipid metabolism and promoting AS. When the endothelium is damaged, LDL can infiltrate into the intima and be oxidized to oxidized low density lipoprotein (ox-LDL), which can be phagocytosed by macrophages and vascular smooth muscle cells (VSMCs) to form foam cells and gradually form atherosclerotic plaques (119, 120). We already know that Hcy can induce ROS accumulation and generate oxidative stress, which can enhance the oxidation of LDL to promote AS (23, 121). Therefore, Hcy can damage the vascular intima and promote the development of AS by affecting the metabolic disorder of lipoproteins.

## 6. Treatment of hyperhomocysteinemia and alleviation of endothelial damage

Due to the vascular damage effect of HHcy and the great harm to the cardiovascular and cerebrovascular, the timely monitoring, reduction and assessment of the blood Hcy level in HHcy patients is a topic worthy of discussion and research. When the plasma Hcy level is found to be elevated, dietary therapy and lifestyle modification can be tried first. Most HHcy is caused by chronic low level of folate and vitamin B12 (122). Correcting the deficiencies of folate and vitamin B12 can reduce Hcy level (123). Fruits, vegetables, and low-fat dairy products are rich in folate and B12 (124), and low-saturated fatty acid and low-fat meals can also reduce Hcy level. In addition, methionine intake should be limited. Another to be introduced is drug therapy, which includes folate, betaine, and vitamin supplements such as vitamin B12 and B6. It is generally accepted that all patients should also be treated with B-complex vitamins to reduce peripheral neuropathy. In terms of mechanism, folate and vitamin B6/B12 can alleviate Hcy-induced ECs apoptosis, oxidative stress, and mitochondrial dysfunction (125), folate also can modulate DNA methylation to delay AS (126). However, the therapeutic challenge is that even though B vitamins reduce Hcy level, which do not seem to reduce the risk of cardiovascular disease (127). The exact mechanism remains to be investigated. One possible explanation is that the beneficial effects of lowering Hcy are offset by the direct adverse effects of B vitamins supplementation (particularly with high-dose folate), or are associated with the pro-inflammatory and pro-proliferative effects of B vitamins on advanced AS lesions (128). It may also be related to the fact that although the plasma Hcy level is reduced, the intracellular Hcy concentration do not change significantly (87). Even so, B vitamins and folate can alleviate the progression of AS by reducing plasma Hcy level and improving epigenetic factors (129). HHcy can also cause extensive damage to a variety of organs, so reducing Hcy is still widely recommended.

In recent years, there have been many studies on alleviating Hcy-induced endothelial damage, which has important implications for the treatment of cardiovascular diseases caused by HHcy. Studies have found that melatonin can protect ECs by alleviating oxidative stress caused by Hcy (3), specifically, melatonin can decrease ROS and lipid peroxidation(LPO) levels induced by Hcy to exert anti-oxidants protection (130). Melatonin also can increase cell migration and downregulate pro-apoptotic proteins caspase-3, caspase-9, cytochrome c (Cyt C), and B-cell lymphoma-2 assaciated X protein (Bax), but upregulate anti-apoptotic protein B-cell lymphoma-2 (Bcl-2) to against Hcy-induced ECs apoptosis (130). Propofol can alleviate inflammation and apoptosis in Hcy-induced human umbilic vein endothelial cells (HUVECs) by inhibiting ERS, manifested as propofol increases cell viability, suppresses NF-κB signaling pathway activation and decreases the expression of inflammatory factors (131). It has been reported that the glucagon-like peptide 1 (GLP-1) analog exendin-4 lowers ERS and enhances protein folding to ameliorate Hcy-induced endothelial dysfunction *in vivo* and *in vitro* (6). Epigallocatechin gallate (EGCG), a well-known anti-oxidant in green tea, can reduce Hcy-induced oxidative damage and apoptosis by enhancing the silent information regulator 1 (SIRT1)/AMP-activated protein kinase (AMPK) survival signaling pathway (132). Estrogen supplementation ameliorates pyroptosis and inflammation in Hcy-treated HUVECs (71), moreover, estradiol-17β can inhibit Hcy mediated damage by promoting H_2_S production *via* upregulating CBS and CSE expression in HUVECs (4). Nicorandil for coronary heart disease alleviates Hcy-induced human coronary artery endothelial cells (HCAECs) dysfunction *via* regulating PI3K/Akt/NOS pathway *in vivo* and *in vitro* (133). L-cystathionine, an amino acid mainly produced during the conversion of methionine to cysteine, which can protect against Hcy-induced mitochondria-dependent apoptosis in HUVECs (75). The lipid-lowering drug atorvastatin can also alleviate ECs damage caused by Hcy (5). To sum up, it is of great significance to further explore the treatment and mechanism of alleviating Hcy-induced endothelial injury for the prevention and treatment of the pro-AS effect of HHcy.

## 7. Conclusion and discussion

As an important pro-AS risk factor, HHcy promotes the occurrence and development of cardiovascular disease. Causes of elevated plasma Hcy level include genetics, nutritional deficiencies, smoking, drugs, demographics, disease states. HHcy damage to the vascular endothelium is the core link of its promotion of AS, and the study of its mechanism is of great significance for the prevention and treatment of HHcy-induced endothelial damage and subsequent AS-related diseases. At present, the mechanisms of Hcy-induced endothelial injury include induction of inflammation and cell death, disturbance of NO production, ROS accumulation and oxidative stress, dysfunction of H_2_S signaling pathway, cellular hypomethylation, protein homocysteinylation, and lipid metabolism disorder. A novel concept is that Hcy-methionine cycle is a metabolic sensor system for methylation-regulated pathological signaling (134). The traditional view is that risk factors such as damage-associated molecular patterns (DAMPs) and pathogen-associated molecular patterns (PAMPs) produce pathological signals by acting on pattern recognition receptors (PRRs) on the cell surface/intracellular/nuclear. However, for metabolism-associated danger signal, probably through receptor-independent recognition by the metabolic sensor system of Hcy-methionine cycle. Further regulating SAM/SAH-dependent methylation in disease conditions and that hypomethylation on frequently modified histone residues may be a key mechanism for cardiovascular disease. This is an important research point of Hcy damage endothelium and cause AS related diseases. In addition, the mechanism of Hcy uptake by ECs is one of the research difficulties. One study found that alanine-serine-cysteine (ASC) transporter systems and lysosome function play an important role in Hcy transport in ECs (135). The specific mechanism needs to be further studied. Exploring more in-depth mechanisms of Hcy damage to the endothelium will help to further discover effective therapeutic targets and methods. The methods of treating HHcy include dietary therapy and supplementation of vitamin B12/B6 and folate. However, a controversial issue is that it has not been proven that lowering Hcy by B vitamins supplementation can reduce the risk of cardiovascular disease. Therefore, the causal relationship between HHcy and B vitamins deficiency and AS remains unclear and requires further investigation. Although lowering Hcy using B vitamins has no beneficial effect on secondary prevention of cardiovascular disease, the role of Hcy in primary disease prevention has not been fully studied. Therefore, more interventions and experimental studies are needed to address the existing knowledge gaps (74). In addition, some studies have found some drugs that can alleviate Hcy-induced endothelial damage by alleviating oxidative stress, ERS, inflammation, such as melatonin, propofol, estrogen, nicorandil, L-cystathionine. In the future, more effective therapeutic drugs need to be further studied. When patients have the following conditions, special attention should be paid Hcy level. Patients with coronary heart disease, cerebrovascular disease or peripheral AS, or with risk factors for cardiovascular and cerebrovascular diseases, such as hypertension, diabetes, obesity, smoking, or a family history of coronary heart disease or AS. In conclusion, the mechanism of Hcy injury to the endothelium and the promotion of AS is a direction worthy of further research, which is of great significance for reducing the occurrence and development of AS-related diseases.

## Author contributions

DY, FC, and XL contributed to the conception, writing, and editing of this manuscript. JC, HL, GZ, JQ, YY, TY, and FP put forward some amendments to the article. All authors contributed to the article and approved the submitted version.

## Glossary

HHCY: hyperhomocysteinemia
AS: atherosclerosis
HCY: homocysteine
ECs: endothelial cells
NO: nitric oxide
ROS: reactive oxygen species
SAM: AdoMet, S-adenosylmethionine
MAT: methionine adenosyltransferase
ATP: adenosine triphosphate
SAH: AdoHcy, S-adenosylhomocysteine
CBS: cystathionine β-synthase
CSE: cystathionine-gamma-lyase
5-methylTHF: 5-methyltetrahydrofolic acid
MS: methionine synthase
THF: tetrahydrofolate
BHMT: betaine-homocysteine methyltransferase
DHFR: dihydrofolate reductase
IL: interleukin
TNF: tumor necrosis factor
NF-κB: nuclear factor kappa-B
PAI-1: plasminogen activator inhibitor-1
SASP: senescence-associated secretory phenotype
NOS: nitric oxide synthase
ADMA: asymmetric dimethylarginine
DDAH: dimethylarginine dimethylaminohydrolase
PKC: protein kinase C
NADPH: nicotinamide adenine dinucleotide phosphate
ERS: endoplasmic reticulum stress
ER: endoplasmic reticulum
Ero-1α: endoplasmic reticulum oxidoreductin-1α
HIF-1α: hypoxia-inducible factor-1α
GPX: glutathione peroxidase
H_2_S: hydrogen sulfide
ERK: extracellular regulated protein kinases
miR: microRNA
LDL: low-density lipoproteins
HDL: high-density lipoproteins
HDL-C: high-density lipoprotein cholesterol
ApoA1: apolipoprotein A1
TG: triglyceride
TC: total cholesterol
LDL-C: low-density lipoprotein cholesterol
ox-LDL: oxidized low density lipoprotein
VSMCs: vascular smooth muscle cells
ICAM-1: intercellular adhesion molecule-1
VCAM-1: vascular adhesion molecule-1
DNMT1: DNA methyltransferase 1
EPCs: endothelial progenitor cells
HTL: homocysteine thiolactone
MetRS: methionyl-tRNA synthetase
PQC: cellular protein quality control
UPR: unfolded protein response
UPS: ubiquitin-proteasome system
ACE: angiotensin-converting enzyme
ANG II: angiotensin II
LPO: lipid peroxidation
Cyt C: cytochrome c
Bax: B-cell lymphoma-2 associated X protein
Bcl-2: B-cell lymphoma-2
HUVECs: human umbilic vein endothelial cells
GLP-1: glucagon-like peptide 1
EGCG: epigallocatechin gallate
SIRT1: silent information regulator 1
AMPK: AMP-activated protein kinase
Nano-Se: nanoscale selenium
HCAECs: human coronary artery endothelial cells
DAMPs: damage-associated molecular patterns
PAMPs: pathogen-associated molecular patterns
PRRs: pattern recognition receptors
ASC: alanine-serine-cysteine.
